# Motor Cortex-Evoked Activity in Reciprocal Muscles Is Modulated by Reward Probability

**DOI:** 10.1371/journal.pone.0090773

**Published:** 2014-03-06

**Authors:** Makoto Suzuki, Hikari Kirimoto, Kazuhiro Sugawara, Mineo Oyama, Sumio Yamada, Jun-ichi Yamamoto, Atsuhiko Matsunaga, Michinari Fukuda, Hideaki Onishi

**Affiliations:** 1 Graduate School of Medical Sciences, Kitasato University, Kanagawa, Japan; 2 Institute for Human Movement and Medical Sciences, Niigata University of Health and Welfare, Niigata, Japan; 3 Graduate School of Medicine, Nagoya University, Nagoya, Japan; 4 Graduate School of Human Relations, Keio University, Tokyo, Japan; University of Regensburg, Germany

## Abstract

Horizontal intracortical projections for agonist and antagonist muscles exist in the primary motor cortex (M1), and reward may induce a reinforcement of transmission efficiency of intracortical circuits. We investigated reward-induced change in M1 excitability for agonist and antagonist muscles. Participants were 8 healthy volunteers. Probabilistic reward tasks comprised 3 conditions of 30 trials each: 30 trials contained 10% reward, 30 trials contained 50% reward, and 30 trials contained 90% reward. Each trial began with a cue (red fixation cross), followed by blue circle for 1 s. The subjects were instructed to perform wrist flexion and press a button with the dorsal aspect of middle finger phalanx as quickly as possible in response to disappearance of the blue circle without looking at their hand or the button. Two seconds after the button press, reward/non-reward stimulus was randomly presented for 2-s duration. The reward stimulus was a picture of Japanese 10-yen coin, and each subject received monetary reward at the end of experiment. Subjects were not informed of the reward probabilities. We delivered transcranial magnetic stimulation of the left M1 at the midpoint between center of gravities of agonist flexor carpi radialis (FCR) and antagonist extensor carpi radialis (ECR) muscles at 2 s after the red fixation cross and 1 s after the reward/non-reward stimuli. Relative motor evoked potential (MEP) amplitudes at 2 s after the red fixation cross were significantly higher for 10% reward probability than for 90% reward probability, whereas relative MEP amplitudes at 1 s after reward/non-reward stimuli were significantly higher for 90% reward probability than for 10% and 50% reward probabilities. These results implied that reward could affect the horizontal intracortical projections in M1 for agonist and antagonist muscles, and M1 excitability including the reward-related circuit before and after reward stimulus could be differently altered by reward probability.

## Introduction

Reward plays an important role in motor learning [1] and in the induction of synaptic plasticity [2]–[6]. In mammals, dopaminergic (DA) neurons in the ventral tegmental area of the substantia nigra respond with increases and decreases in their firing rate as a consequence of rewarding stimuli [7]. Among the areas potentially influencing the primary motor cortex (M1), many are involved in reward processing, including the substantia nigra and striatum [1], [8]–[12]. Recent retrograde tracing research found about 70% of DA midbrain neurons projecting to M1 were located in the ventral tegmental area [13]. In M1, DA terminals are distributed inhomogeneously with a preference for deep cortical layers V and VI [14]. Regarding postsynaptic elements, D1 and D2 receptors are expressed in both superficial (I, II, and III) and deep (V and VI) layers [15]. In addition, animal experimentation has suggested that extensive, horizontally oriented, intrinsic axon collaterals in layers III and V provide inputs to many different movement representations in M1 [16]. In human experimentation, the output from the common M1 site may diverge onto agonist and antagonist muscles with different “gain” according to the final movement to be performed, presumably regulated by the horizontal intracortical projections interconnecting functionally related neuronal clusters within M1 [17]. DA neurons may play a significant role in this context. Recent studies [14] revealed that dopamine modulates M1 circuitry by affecting various processes of motor learning-dependent plasticity. Motor skill learning induces a long-lasting increase in synaptic strength in M1 horizontal connections of layers II/III suggesting an association with long-term potentiation (LTP)-like plasticity [18], [19]. The D1-receptor antagonist SCH29339 and the D2-receptor antagonist raclopride markedly reduced the ability of M1 horizontal connections to form LTP [6]. These results would suggest that intact DA signaling is necessary for synaptic plasticity in M1 and reward information may influence motor behavior by modulating the excitability of the M1 to diverge onto agonist and antagonist muscles.

Because the corticomotoneuron system can be activated by transcranial magnetic stimulation (TMS), there is a suggestion that the change of motor evoked potentials (MEPs) depends on the M1 activity [20]. Previous studies showed changes in MEP amplitudes just prior to [21] and after [12], [22] voluntary movements in response to reward stimulus. These results would suggest that reward information may influence the excitability of the M1. Because the time resolution of TMS is suitable for observing changes in M1 excitability responding to reward stimulus, we considered that changes in MEPs would be observed by this technique just before and after voluntary movements in response to reward stimulus. However, although the relation between reward and reciprocal inhibition is crucial for human movements, change in cortical circuits for reciprocal muscles by reward probability is unknown. If the horizontal intracortical projections for reciprocal muscles exist within M1 and reward induces a reinforcement of transmission efficiency of intracortical circuits, intracortical circuits for reciprocal muscles might be changed by reward. To understand reward-induced change in M1 excitability for reciprocal muscles, we investigated the relation between reward probability and M1 excitability for reciprocal muscles. On the basis of background information on reward and reciprocal inhibition, we hypothesized that M1 excitability for reciprocal muscles is changed in reference to reward probability. To test this hypothesis, we investigated the excitatory system within the human M1 by using TMS during the performance of probabilistic reward tasks.

## Materials and Methods

### Subjects

In a preliminary experiment, the average values and standard deviations (SD) of peak-to-peak MEP amplitudes of the right FCR muscle in 4 healthy and neurologically intact people (2 men and 2 women aged 21–22 years) were assessed to determine the sample size. The coil was placed tangentially to the scalp and was held with the handle pointing backward and sideways, approximately 45° to the midline, to induce a current in the left brain from posterior-lateral to anterior-medial. The resting motor threshold (RMT) was determined as the minimum stimulus intensity required to produce a MEP in the relaxed FCR muscle of at least 50 μV in 5 of 10 consecutive trials. We recorded the MEPs evoked by 10 stimulations at 120% of the RMT (interstimulus interval was 5 s). The MEP amplitudes of ranged from 0.17 to 1.63 mV (mean ± SD, 0.45±0.27 mV). Gupta and Aron [22] investigated the difference in MEP amplitudes between reward (strongly and weakly desired) and non-reward (neutral) conditions. Their results noted that mean delta MEP amplitude between reward and non-reward conditions was 0.10 mV (strongly desired condition, 0.98 mV; weakly desired condition, 0.85 mV; neutral condition, 0.81 mV). Therefore, standard effect size (0.40) was calculated based on the mean and SD of MEP amplitudes in our preliminary experiment and an expected 0.10 mV difference in the MEP amplitudes. Subsequently, the sample size calculation was based on a desired 95% statistical power to detect a 0.10-mV difference in the MEP amplitudes, with a 2-sided α of 1%. A sample size of 223 MEPs was derived by insertion of 1-power (0.01), β (0.05), and standard effect size (0.40) values in the Hulley matrix [23]. Previous experiments using reward tasks [12], [21], [22] recorded 18 to 60 MEP amplitudes in each condition to detect the change in MEP amplitude according to the reward. We therefore took the variability of MEP amplitudes into consideration and planned to recruit 8 subjects (30 MEPs per each condition for each subject) for this study. This, the participants comprised 8 healthy and neurologically intact right-handed volunteers (4 men and 4 women) aged 21–29 (mean ± SD, 22.0±2.8) years. They were naïve as to the purpose of the experiment and were screened for potential risk of adverse events during TMS [24]. Written informed consent was obtained from all subjects prior to their participation. They did not take any medications and did not have any neurological or psychiatric diseases. Handedness was determined by the Edinburgh Handedness Inventory [25]. The mean laterality quotient score was 1.0±0.0 (mean ± SD) points. The experimental procedures were approved by the Ethics Committee of Niigata University of Health and Welfare. This study was performed in accordance with the Declaration of Helsinki.

### Electromyographic recordings

**Figure 1:**
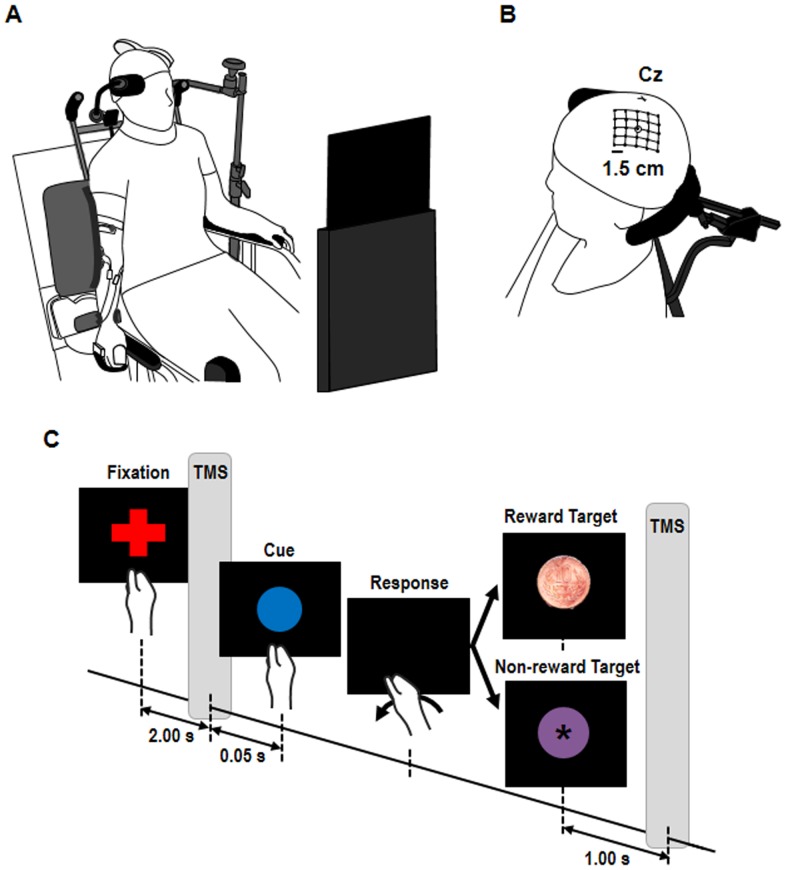
Experimental setup. (**A**) Change in primary motor cortex (M1) excitability for agonist and antagonist muscles during probabilistic reward tasks was investigated. Subjects were seated comfortably in a chair. The right arm hung to the side in a relaxed posture, with the palm and forearm placed on the equipment. (**B**) Schematic of a head with a grid showing the stimulated scalp sites. Cz represents the intersection of nasion-inion and the interaural lines. (**C**) Experimental design in probabilistic reward task. Probabilistic reward tasks comprised 3 conditions of 30 trials: 30 trials contained 10% reward stimulus and the remaining trials contained a non-target stimulus, 30 trials contained 50% reward stimulus, and 30 trials contained 90% reward stimulus. The inter-trial interval was randomized between 7–8 s. Single-pulse transcranial magnetic stimulation (TMS) was delivered at 2 s after appearance of the red fixation cross and 1 s after appearance of the reward/non-reward stimuli.

Subjects were comfortably seated in front of a 10.1-inch screen placed 50 cm from the subject's eyeline (Figure 1A). The right arm hung to the side in a relaxed posture with the palm and forearm placed on the equipment. The subject's forearm was fixed by a cushioned support made of particle-foam plastic, and the hand was inserted in a hand-piece with the fingers (excluding thumb) held by a strap in the flexion position. The wrist was left entirely free to perform flexion movements, and the equipment automatically returned to the start position (wrist neutral posture) after wrist flexion. The left arm rested on the subject's thigh and was kept relaxed.

Prior to electromyography (EMG) recording, the skin overlying the FCR and ECR muscles was cleaned with alcohol to reduce its electrical resistance. The FCR and ECR muscle bellies were identified by palpation during manually resisted wrist flexion and extension, respectively. The FCR electrodes were placed ventrally on the forearm approximately 7 cm distal from the medial epicondyle, and ECR recording electrodes were placed dorsally on the forearm approximately 3 cm distal from the lateral epicondyle [26]. For the ground, a rectangular electrode band was wrapped around the upper extremity approximately 5 cm proximal to the elbow. Surface EMG activity was recorded from the FCR and ECR muscles by means of disposable, self-adhesive Ag-AgCl electrodes (diameter, 2 cm). The centers of the electrodes were placed 2 cm apart over the middle portion of the muscle belly and were aligned longitudinally with the muscle fiber direction in accordance with previous studies [26]–[28]. The EMG signals were amplified (×100) and bandpass filtered (5–2000 Hz) with an amplifier (DL-140, 4Assisr, Tokyo, Japan). Then, the EMG data were digitized at 10 kHz (PowerLab; ADInstruments, Colorado Springs, CO, USA) and stored on magnetic media for later retrieval and off-line analysis.

### Transcranial magnetic stimulation

Two Magstim 200^2^ stimulators connected through a BiStim unit (Magstim Co., Ltd., Whitland, Dyfed, UK) were used for TMS, which was delivered to the scalp surface through a figure-of-eight coil (internal diameter of each wing was 70 mm) with a monophasic current waveform. A tight-fitting cap was placed over the participant's head. The intersection of nasion-inion and the interaural lines were drawn on the cap using a marker pencil to localize the vertex according to the 10–20 International System. The coil was placed tangentially to the scalp and was held with the handle pointing backward and sideways, approximately 45° to the midline, to induce a current in the left brain from posterior-lateral to anterior-medial. At the start of the experiment, the optimal coil position for eliciting the maximum MEPs in the FCR or ECR muscles (the so-called hot spot) was marked with a soft-tipped pen, respectively. The hotspots were found by moving the coil over the left motor cortex to find the site that elicited the MEP with the largest amplitude in the muscle of interest [29]. The RMT at the hot spot was determined as the minimum stimulus intensity required to produce a MEP in the relaxed FCR or ECR muscles of at least 50 μV in 5 of 10 consecutive trials, respectively. The stimulus intensity was altered in 1% increments of maximum stimulator output throughout this process.

### Motor representational map

To map out the muscle representation, a 25-position grid (6×6 cm) was marked on each cap, and its center was on the hot spot of the FCR or ECR muscles, respectively (Figure 1B). For each scalp position, we recorded the MEPs evoked by 5 stimulations at 120% of the RMT in a clockwise spiral course, beginning at the hot spot of the FCR and ECR muscles, respectively (interstimulus interval was 5 s). The figure-of-eight-shaped coils used in this study were more focal, producing maximal current at the intersection of the two round components [30]. Therefore, the intersection of the two round components conformed to each position grid. The map areas corresponded to the stimulated positions. The center of gravity (CoG) of each muscle was computed separately as a measure of the amplitude-weighted center of the motor representational map [31]–[33]. It was expressed as a bivariate measurement with an anteroposterior (*x*) and mediolateral (*y*) coordinate, using the following formula: CoG  =  [∑*a_i_x_i_*/∑*a_i_*, ∑*a_i_y_i_*/∑*a_i_*], where *x_i_*, *y_i_* were stimulation position coordinates and *a_i_* was amplitude. The CoGs corresponded to the locations of the most excitable populations of neurons that project to the target muscles. Cortical excitability recordings were performed at the midpoint between CoGs of the FCR and ECR muscles, respectively, because the input-output curves measured at the midpoint between the CoGs of the FCR and the ECR muscles and the CoG of each muscle were homogenous [33]. In input-output curves, the relationship between MEP amplitude and TMS intensity is typically non-linear, with a steep increase above the motor threshold and a plateau phase at high intensities [33]–[36]. The sigmoidal shape of the input-output curve was found due to a combination of the following factors: the way cortical elements were recruited by the TMS; the combination of multiple components of the corticospinal volley; the recruitment of motor neurons with progressively larger motor unit potentials; and the synchronization of single motor unit discharges [34]. Thus, the characteristics of recruitment of motor neurons and corticospinal neurons appear to influence the input-output curve. Therefore, the homogeneity of the input-output curve [33] implies that cortical excitability recordings at the midpoint of CoGs between reciprocal muscles could be an alternative for the separate cortical excitability recordings by stimulating each reciprocal muscle.

### Cortical excitability recordings

Peak-to-peak MEP amplitudes during probabilistic reward tasks were recorded. In addition, measures of motor cortical excitability using TMS included RMT, unconditioned MEP, short-interval intracortical inhibition (SICI), and short-latency afferent inhibition (SAI) before and after the probabilistic reward task for the FCR or ECR muscle under each respective reward probability condition [37]. For the measurement of SICI, paired pulse magnetic stimuli were applied [37]. The intensity of the conditioning stimulus was adjusted to 80% of RMT, and that of the test stimulus was adjusted to 120% of RMT with an interstimulus interval of 3 ms [12], [38]. For the measurement of SAI, transcutaneous electrical stimulation was delivered via bipolar surface electrodes arranged anode and cathode in line (the cathode was 2 cm proximal to the anode) over the median nerve at the wrist to elicit the abductor pollicis brevis muscle contraction. After the appropriate stimulation site was determined, a conditioning constant-current square-wave electrical pulse of 0.2-ms duration was applied to the median nerve at the wrist, with the cathode placed proximally, at the intensity of the motor threshold for evoking just visible muscle contraction in the abductor pollicis brevis muscle [39]. The test stimulus was given at an interstimulus interval of 20 ms after the conditioning pulse over the contralateral M1 [12], [40]. We recorded 10 iterations of each of unconditioned MEP, SICI, and SAI trial with a frequency of 0.2 Hz in a randomized order before and after the probabilistic reward task. Intracortical inhibitions as the ratio of conditioned to unconditioned MEP were calculated.

### Experimental task

The probabilistic reward task was applied, as reported previously [12], [21], [22], [41]–[43]. Each trial began with a cue (red fixation cross) displayed on the screen for 2.05 s, followed by display of a blue circle for 1 s (Figure 1C). The subject was instructed to perform wrist flexion and press a button with the dorsal aspect of the middle finger phalanx as quickly as possible in response to the disappearance of the blue circle without looking at his/her hand or the button. There was no need for the subjects to view their hand and the button because the button was always pressed by wrist flexion. Two seconds after the button press, the reward/non-reward stimulus was randomly presented for 2-s duration as feedback for the subject. In previous reward tasks [12], [21], [22], money in the amount of 10 to 500 Japanese yen (about $0.10 to $5) was used as reward. Thus, in our study, the reward stimulus was a picture of a Japanese 10-yen coin, which had a rewarding value as it represented an actual momentary monetary reward. The non-reward stimulus was a mauve circle containing an asterisk sign (*), and this stimulus represented a target without a rewarding value, to control attention and other sensorimotor effects [12]. Previous experiments using reward tasks [12], [22] recorded 18 to 60 trials per condition for within- and between-subjects comparison. Therefore, the probabilistic reward task comprised 3 conditions of 30 trials per condition: 30 trials contained a 10% reward stimulus and the remaining trials contained a non-target stimulus, 30 trials contained a 50% reward stimulus, and 30 trials contained a 90% reward stimulus. The order of conditions of the three reward probabilities was randomized considering a counterbalance. Reward probabilities and order of conditions were not revealed to the subjects. The inter-trial interval was randomized between 7–8 s. The probabilities of the reward stimulus were predetermined. We delivered single-pulse TMS at 2 s after the appearance of the red fixation cross and 1 s after the reward/non-reward stimuli [12], [21], [22]. Reaction time was calculated as the time elapsed between disappearance of the blue circle and the button press. Before the start of the experiment, a familiarization session was performed to allow subjects to understand the experimental protocol. The familiarization session comprised 30 trials containing a 100% reward stimulus. Each subject received an actual monetary reward at the end of the experiment.

### Data analysis

Every single MEP was visually inspected, and MEPs contained the pre-stimulus background EMG were discarded. This ensured the removal of data that may have been contaminated with low-level motoneuronal activity by recrementitious body movements [44]. Although MEPs in the wrist muscles are predominantly polyphasic [20], [30], the results focused on change in M1 excitability for reciprocal muscles in reference to reward probability. We therefore used peak-to-peak amplitude of the MEPs. For quick movements, healthy young individuals produce net torque at a joint by optimally scaling the activation of the agonist and the concurrent activation of the antagonist muscles [45], [46]. Although activation of the agonist contributes to faster movement, one function of the antagonist burst appears to be to provide a braking force to stop the limb [47], [48]. However, the onset of an antagonist burst as a braking force will occur during the initial acceleration phase of movement because it leads to a decrease in the velocity of movement. Therefore, agonist and antagonist EMG activities for fast movement may be observed as a result of offsetting the facilitation of agonist activity and the inhibition of antagonist activity. This is especially important at the onset of fast contractions where there is inadvertent activation of the antagonist muscle. Therefore, relative MEP amplitudes were calculated as the ratio of peak-to-peak MEP amplitudes of FCR muscle to ECR muscle. Repeated measures analysis of variance (ANOVA) was performed to compare differences in MEP amplitudes and reaction time during the probabilistic reward task between 3 different reward probabilities (10%, 50%, and 90%). Two-tailed paired *t*-test with Bonferroni correction was used for post hoc analysis. In addition, differences in RMT, relative MEP, SICI, and SAI before and after the probabilistic reward task were analyzed by the paired *t*-test. All data are expressed as mean ± standard error of the mean (SEM). A *P* value of less than 0.05 was considered statistically significant. All statistical procedures were carried out with PASW Statistics 18 software (IBM, New York, NY, USA).

## Results

All subjects completed all experimental conditions. None of the subjects experienced any side effects from TMS during the experiments.

### Motor representational map

**Figure 2:**
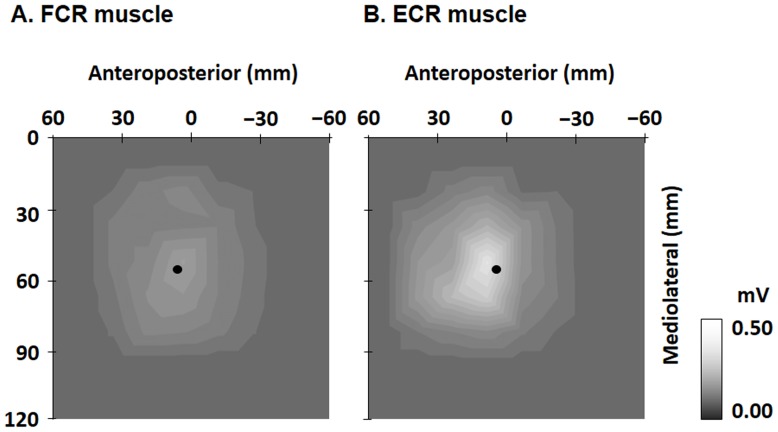
Two-dimensional maps. The color code of each map of FCR (A) and ECR (B) muscles ranges from gray (0 mV) to white (0.5 mV or over). The map areas of the FCR and ECR muscles clearly overlapped, although they were spread differently. The center of gravity (black circle) of the FCR muscle was located at *x* (anteroposterior)  = 6.5±2.6 mm and *y* (mediolateral)  = 56.5±2.3 mm and that of the ECR muscle was located at *x* = 4.5±3.6 mm and *y* = 56.4±2.7 mm. FCR: flexor carpi radialis; ECR: extensor carpi radialis.

The RMTs of the FCR and ECR muscles were 46.0±1.6% and 43.6±5.0% of the maximum stimulator output, respectively. Map areas for the FCR and ECR muscles are shown in Figure 2. The reciprocal muscle areas clearly overlapped, although they were not identical. The CoG of the FCR was more laterally located than that of the ECR in 5 of 8 subjects. The CoG of the FCR was located at *x* (anteroposterior)  = 6.5±2.6 mm and *y* (mediolateral)  = 56.5±2.3 mm, and that of the ECR was at *x* = 4.5±3.6 mm and *y*  = 56.4±2.7 mm. The midpoint between the CoGs of the FCR and ECR muscles was located at *x* = 5.3±3.0 mm and *y* = 56.4±2.3 mm.

### Cortical excitability

**Figure 3:**
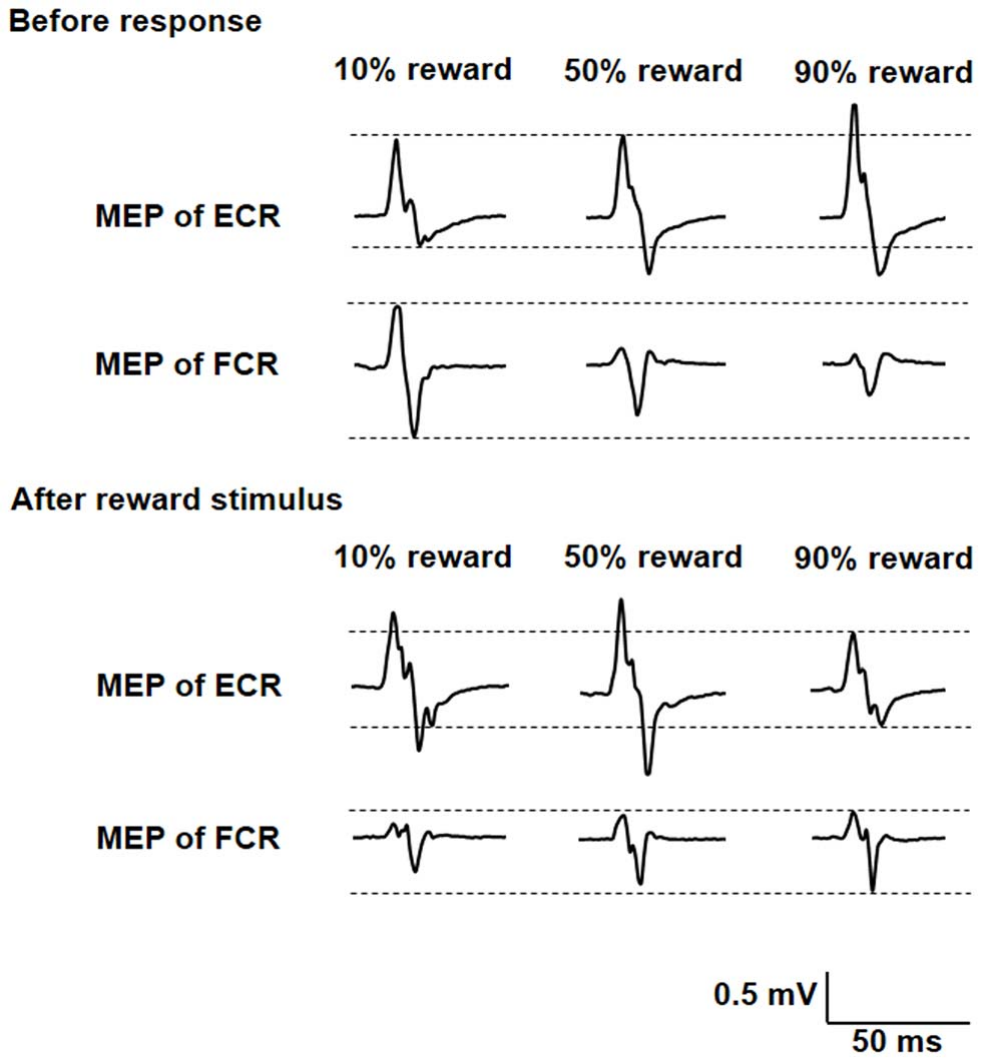
Electromyography traces of the right FCR and ECR muscles in one representative subject. MEP amplitude of the FCR muscle at 2 s before response was the highest for 10% reward probability during the task, whereas that of the ECR muscle was the lowest for 10% reward probability. However, MEP amplitude of the FCR muscle at 1 s before response was the highest for 90% reward probability during the task, whereas that of the ECR muscle was the lowest for 90% reward probability. MEP, motor-evoked potential; FCR, flexor carpi radialis; ECR, extensor carpi radialis.

The EMG traces of the right FCR and ECR muscles in one representative subject during the probabilistic reward task are shown in Figure 3. Peak-to-peak MEP amplitude of the FCR muscle at 2 s after the red fixation cross was the highest for 10% reward probability, whereas that of the ECR muscle was the lowest for 10% reward probability (top 2 rows). However, peak-to-peak MEP amplitude of FCR muscle at 1 s after reward stimuli was the highest for 90% reward probability, whereas that of the ECR muscle was the lowest for 90% reward probability (bottom 2 rows).

**Figure 4:**
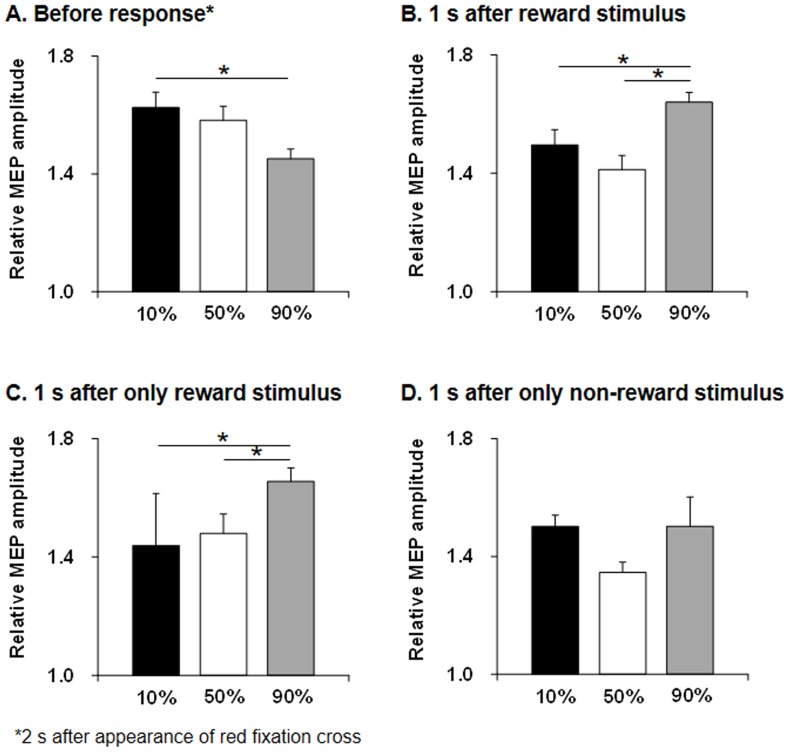
Bar graphs of relative MEP amplitudes for FCR and ECR muscles. Relative MEP amplitude at 2(**A**) and at 1 s after reward/non-reward stimuli (**B**) during the task. Relative MEP amplitude at 2 s after the red fixation cross was significantly higher for 10% reward probability than for 90% reward probability (*p* = 0.008) during the task, whereas relative MEP amplitude at 1 s after reward/non-reward stimuli was significantly higher for 90% reward probability than for 10% (*p* = 0.001) and 50% (*p* = 0.001) reward probabilities. Bar graphs of relative MEP amplitudes for FCR and ECR muscles at 1 s after only reward stimuli presentation (**C**) and only non-reward stimuli presentation (**D**) during the task. Relative MEP amplitude at 1 s after only reward stimuli presentation was significantly higher for 90% reward probability than for 10% (*p*<0.0001) and 50% (*p* = 0.006) reward probabilities. However, relative MEP amplitudes for FCR and ECR muscles at 1 s after only non-reward stimuli presentation were not significantly changed. MEP, motor-evoked potential; FCR, flexor carpi radialis; ECR, extensor carpi radialis.

Relative MEP amplitudes for the FCR to ECR muscles during probabilistic reward tasks are shown in Figure 4 (A, B) and Table 1. Use of repeated measures ANOVA revealed a significant difference of probabilities at 2 s after the red fixation cross (F = 4.153, *p* = 0.016) and at 1 s after reward/non-reward stimuli (F = 1.86, *p*<0.0001). Post hoc testing showed relative MEP amplitude at 2 s after the red fixation cross was significantly higher for 10% reward probability than for 90% reward probability (*p* = 0.008), whereas relative MEP amplitude at 1 s after reward/non-reward stimuli was significantly higher for 90% reward probability than for 10% (*p* = 0.001) and 50% (*p* = 0.001) reward probabilities. Relative MEP amplitudes for the FCR and ECR muscles at 1 s after only reward stimulus presentation are shown in Figure 4C and Table 2. Use of repeated measures ANOVA revealed a significant difference of probabilities at 1 s after reward stimuli (F = 12.98, *p*<0.0001). Post hoc testing showed relative MEP amplitude at 1 s after reward stimuli was significantly higher for 90% reward probability than for 10% (*p*<0.0001) and 50% (*p* = 0.006) reward probabilities. However, relative MEP amplitudes for FCR and ECR muscles at 1 s after only non-reward stimuli presentation were not significantly changed (*p* = 0.225) (Figure 4D and Table 2).

**Table 1:** Peak-to-peak MEP amplitudes obtained for the ECR and FCR muscles during probabilistic reward tasks.

	Reward probability			
MEP amplitudes	10%	50%	90%	P*
Before reaction				
Relative amplitude (FCR/ECR)	1.63±0.05	1.58±0.05	1.45±0.03	0.016
FCR muscle (mV)	1.32±0.03	1.11±0.03	1.16±0.02	
ECR muscle (mV)	1.01±0.02	0.91±0.02	1.00±0.02	
After reward stimulus				
Relative amplitude (FCR/ECR)	1.50±0.04	1.41±0.03	1.64±0.04	<0.0001
FCR muscle (mV)	1.52±0.03	1.40±0.03	1.42±0.03	
ECR muscle (mV)	1.28±0.03	1.33±0.04	1.15±0.03	

Values are mean ± standard error of the mean. MEP, motor-evoked potential; ECR, extensor carpi radialis; FCR, flexor carpi radialis.

*Differences in MEP amplitudes between reward probabilities were analyzed by repeated measures analysis of variance.

**Table 2:** Peak-to-peak MEP amplitudes obtained for the ECR and FCR muscles after reward and non-reward stimuli presentations during probabilistic reward tasks.

	Reward probability			
MEP amplitudes	10%	50%	90%	P*
Reward stimulus				
Relative amplitude (FCR/ECR)	1.44±0.18	1.48±0.07	1.66±0.04	<0.0001
FCR muscle (mV)	1.51±0.03	1.46±0.05	1.42±0.03	
ECR muscle (mV)	1.31±0.11	1.29±0.04	1.13±0.08	
Non-reward stimulus				
Relative amplitude (FCR/ECR)	1.50±0.04	1.35±0.03	1.50±0.10	0.225
FCR muscle (mV)	1.52±0.03	1.34±0.04	1.39±0.04	
ECR muscle (mV)	1.28±0.04	1.37±0.06	1.26±0.08	

Values are mean ± standard error of the mean. MEP, motor-evoked potential; ECR, extensor carpi radialis; FCR, flexor carpi radialis.

*Differences in MEP amplitudes between reward probabilities were analyzed by repeated measures analysis of variance.

**Figure 5:**
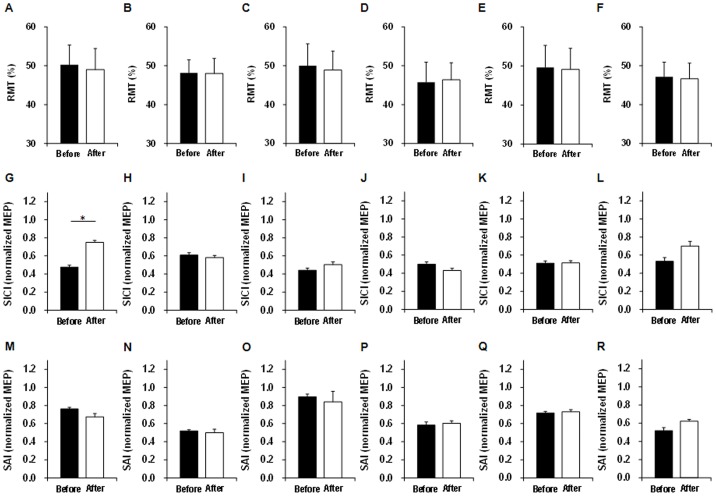
Bar graphs of RMT, SICI, and SAI before and after probabilistic reward tasks. RMT of FCR (**A**) and ECR (**B**) for 10% reward probability, RMT of FCR (**C**) and ECR (**D**) for 50% reward probability, RMT of FCR (**E**) and ECR (**F**) for 90% reward probability, SICI of FCR (**G**) and ECR (**H**) for 10% reward probability, SICI of FCR (**I**) and ECR (**J**) for 50% reward probability, SICI of FCR (**K**) and ECR (**L**) for 90% reward probability, SAI of FCR (**M**) and ECR (**N**) for 10% reward probability, SAI of FCR (**O**) and ECR (**P**) for 50% reward probability, and SAI of FCR (**Q**) and ECR (**R**) for 90% reward probability. Only the SICI of the FCR was significantly decreased after 10% probabilistic reward tasks (*p = *0.0008). RMT, resting motor threshold; SICI, short-interval intracortical inhibition; SAI, short-latency afferent inhibition; FCR, flexor carpi radialis; ECR, extensor carpi radialis.

Differences in RMT, relative MEP, SICI, and SAI before and after probabilistic reward tasks are shown in Table 3 and Figure 5. The changes in RMT of the FCR and ECR, relative MEP, SICI of the FCR and ECR, and SAI of the FCR and ECR for 10%, 50%, and 90% reward probabilities were small and were not significantly different before and after probabilistic reward tasks. However, SICI of the FCR was significantly decreased after 10% probabilistic reward tasks (*p* = 0.0008).

**Table 3:** MEP amplitudes and values of RMT, SICI, and SAI obtained for the ECR and FCR muscles before and after probabilistic reward tasks.

	FCR muscle (mV)								
	10% reward probability			50% reward probability			90% reward probability		
	Before	After	P*	Before	After	P*	Before	After	P*
RMT (%)	50.3±5.1	49.0±5.4	0.072	50.0±5.6	48.9±4.9	0.301	49.6±5.6	49.1±5.4	0.590
MEP (mV)	0.28±0.01	0.33±0.01	0.260	0.27±0.01	0.23±0.01	0.115	0.29±0.01	0.29±0.01	0.816
SICI (conditioned/test MEP)	0.48±0.02	0.75±0.02	0.008	0.44±0.02	0.50±0.03	0.314	0.51±0.02	0.51±0.04	0.954
SAI (conditioned/test MEP)	0.76±0.02	0.68±0.04	0.238	0.90±0.03	0.84±0.12	0.720	0.72±0.02	0.73±0.02	0.884

	ECR muscle (mV)								
	10% reward probability			50% reward probability			90% reward probability		
	Before	After	P*	Before	After	P*	Before	After	P*
RMT (%)	48.1±3.4	48.0±3.9	0.732	45.88±5.1	46.4±4.4	0.760	47.1±3.8	46.6±4.0	0.699
MEP (mV)	0.39±0.02	0.46±0.03	0.201	0.36±0.01	0.43±0.02	0.143	0.42±0.04	0.44±0.01	0.649
SICI (conditioned/test MEP)	0.61±0.03	0.58±0.02	0.796	0.50±0.03	0.43±0.02	0.316	0.53±0.04	0.70±0.05	0.109
SAI (conditioned/test MEP)	0.52±0.01	0.50±0.04	0.792	0.58±0.04	0.60±0.03	0.826	0.52±0.03	0.62±0.02	0.179

Values are mean ± standard error of the mean. MEP, motor-evoked potential; ECR, extensor carpi radialis; FCR, flexor carpi radialis; RMT, resting motor threshold; SICI, short-interval intracortical inhibition; SAI, short-latency afferent inhibition.

*Differences in MEP amplitudes before and after probabilistic reward task were analyzed by paired t-tests.

### Behavioral results

The mean reaction time was 913.3±17.3 ms for 10% reward probability, 931.8±14.7 ms for 50% probability, and 874.1±14.3 ms for 90% reward probability. Repeated measures ANOVA revealed no significant difference in reaction time between reward probabilities (F = 0.810, *p* = 0.446). Each subject received a total of 750 Japanese yen (about $7.5) at the end of the experiment.

## Discussion

In the present study, we observed a change in M1 excitability for reciprocal muscles during the performance of probabilistic reward tasks. The results of this study indicated that (a) relative MEP amplitudes of agonist (FCR) and antagonist (ECR) muscles before reward stimulus were highest for 10% reward probability during probabilistic reward tasks, (b) relative MEP amplitudes of agonist and antagonist muscles after reward stimulus presentation were highest for 90% reward probability during probabilistic reward tasks, (c) relative MEP amplitudes of agonist and antagonist muscles after non-reward stimulus presentation were not changed during probabilistic reward tasks, and (d) SICI of the agonist muscle was decreased after 10% probabilistic reward tasks. These systematic observations provided evidence that M1 excitability for reciprocal muscles was affected by reward probability. To our knowledge, this is the first systematic study to demonstrate a change in M1 excitability for reciprocal muscles during the performance of probabilistic reward tasks.

Many areas influence M1 in reward processing, e.g., the ventral tegmental area, striatum, prefrontal and orbitofrontal cortex, amygdala, anterior cingulate cortex, supplementary motor area, nucleus accumbens, and the hippocampus [1], [8]–[10], [49]–[52]. The activities of these neurons increase or decrease in response to reward or non-reward [7], which is believed to improve behavioral outcome by strengthening both circuits implicated in successful actions accompanying reward stimulus. Kapogiannis et al. [21] showed that reward expectation altered M1 excitability induced by TMS in a reward task that was simulated by a slot machine. They suggested that an excitability change in M1 was associated with the expectation of reward and modified by prior experience. Gupta and Aron [22] found that stimuli that were more strongly desired elicited an increase in M1 excitability induced by TMS as compared with less desired or neutral stimuli. Thabit et al. [12] showed that the excitability changes in M1 were induced by a momentary reward and suggested that this might be related to the reward-related motor activity at the cortical level or may reflect its occurrence at the striatal level. Collectively, these three studies suggest that reward signals modulate motor output in the cortex and that MEPs can be used as objective correlates of motivation, at least in controlled experimental settings.

The first additional new observation in our study was that relative MEP amplitudes of agonist and antagonist muscles before reward stimulus during the probabilistic reward task were highest for 10% reward probability. Ten percent reward probability also means that reward was not presented in 90% of the trials. Interestingly, the result that the highest relative MEP amplitude occurred at a 10% reward (90% non-reward) probability could lead to a counterintuitive prediction because this result may reflect on highest reward expectation at the lowest reward probability (high non-reward probability). Previous animal experimentation noted that DA neuron activity coded for relative outcome in light of the anticipation that is generated on the basis of previous experience [7]. If the result is better than expected (i.e., in the case of a positive reward prediction error), the firing rate of these neurons will increase. In contrast, outcomes that do not meet expectations (a negative reward prediction error) decrease the activity of these neurons. In human experimentation, Michael [53] noted that deprivation of reward stimulus momentarily increased the reinforcing effectiveness. In addition, Gottschalk et al. [54] examined the effects of deprivation on the approach behavior for food. Their results demonstrated that deprivation increased the approach behavior. These results imply that the expectation of reward associated with the reward prediction error cannot be maximized under the highest uncertainty condition (50% reward probability) in humans. One possible explanation for the highest relative MEP amplitudes of 10% reward probability before reward stimulus is that 10% reward probability might induce the highest reward expectation under the low reward probability related to deprivation of reward. However, Thabit et al. [12] noted that the MEP amplitude did not show any significant changes for reward vs. non-reward stimulus. In marked contrast to the findings of Thabit et al. [12], in the present study, relative MEP amplitudes of agonist and antagonist muscles after reward stimulus were highest for 90% reward probability. The Thabit et al. study observed the peak-to-peak MEP amplitude of only the agonist muscle. In contrast, relative MEP amplitude was calculated as the ratio of peak-to-peak MEP amplitudes of agonist muscle to antagonist muscle in the present study. Although such activation of the agonist contributes to faster movement, the onset of an antagonist burst exerts a braking force [47], [48]. Therefore, the activation of the agonist and the concurrent activation of the antagonist muscles have to be optimally scaled for quick movements [45], [46]. As a result, the difference in relative MEP amplitudes between reward probabilities may be exposed in our study.

Previous studies suggested that Ia inhibitory interneurons are facilitated by the corticospinal tract or inhibitory volleys that descend from M1 to the motor neuron of the antagonist muscle [33], [44], [55], [56]. Horizontal intrinsic axon collaterals in layers III and V provide inputs to many different forelimb movement representations in M1 in animals [16]. Dopamine may affect the horizontal intracortical projections in layers III and V within M1. Recent studies revealed that the integrity of DA fibers in M1 is a prerequisite for successful acquisition of motor skills [18], and most DA fibers innervating M1 originate within the midbrain [13]. At the level of synapses, a long-lasting increase in synaptic strength of the horizontal connections in layers II/III in M1 can be induced by motor skill learning, indicating a possible association with LTP-like plasticity [18]. Several weeks after skill acquisition, the ability to form LTP is restored and the horizontal connections of layers II/III remain strengthened [19]. In line with this assumption, dopamine modulates cortical activity by enhancing transmission at active synapses while suppressing it at inactive ones [57]. In the present study, the optimal coil position for simultaneously eliciting MEPs from reciprocal muscles was determined systematically. Thereby, TMS could simultaneously stimulate reciprocal muscles. This was thought to be the basis from the observation of M1 excitability for reciprocal muscles during probabilistic reward tasks. One possible explanation for the changes in relative MEP amplitudes of agonist and antagonist muscles is that the effect of dopamine release in the vicinity of highly active cortical synapses could be to increase the transmission efficiency by strengthening the synapses for the agonist muscle while suppressing it for the synapses of the antagonist muscle. However, our study was not able to directly observe the change in dopamine release. Koepp et al. [58] used ^11^C-labelled raclopride and positron emission tomography (PET) scans to provide evidence that endogenous dopamine was released in the human striatum during a goal-directed behavioral task. Zald et al. [59] reported on ^11^C-labelled raclopride PET studies in which healthy humans performed card selection tasks for monetary rewards. They noted that relative to the sensorimotor control condition, the reward schedules produced increases in dopamine transmission. Therefore, further research is needed to investigate the effect of dopamine release on reciprocal inhibition function using both TMS and brain imaging methods.

Our study showed relative MEP amplitudes of agonist and antagonist muscles after reward presentation were highest for 90% reward probability. Collectively, relative MEP amplitudes for agonist and antagonist muscles at 1 s after only reward stimulus presentation were higher for 90% reward probability than for 10% and 50% reward probabilities, whereas relative MEP amplitudes after non-reward stimulus presentation were not changed. This is the second additional new observation from our study. The phasic burst firing of DA neurons was found to be higher in response to unpredicted or under-predicted rewards [60]. Schultz et al. [7] indicated that most DA neurons showed a short burst of impulses in reference to the reward itself before training and in the initial phases of training for a few days. This phasic activation of the midbrain DA neurons causes a rise in the dopamine concentration of the striatum. Wickens et al. [1] suggested that reward-related dopamine pulses released in the striatum are proposed to facilitate the selection of particular pathways through the basal ganglia to the M1, and hence of particular actions, according to past and anticipated rewards. Although we could not identify the exact mechanism, M1 excitability increased by 90% reward probability in reference to the maximum global “reward” signal, indicating that the 3 conditions of 30 trials comprising our probabilistic reward task might correspond to the initial phases of learning. To find more answers, future studies should consider the time course of change in M1 excitability in relation to the long-term learning process of reward probabilities.

Some studies have shown that changes in SICI and SAI are inversely related [61]–[64], and recently, a model of two distinct reciprocally connected subtypes of GABA inhibitory interneurons with convergent projections onto the corticospinal neurons was suggested to explain this inverse relation [64]. Dopamine neurons excite GABAergic interneurons [65], which inhibit cortical pyramidal cells [15]. Accordingly, dopamine release in the striatum may affect SAI in M1 indirectly. Thabit et al. [12] found that SICI was increased and SAI was decreased in response to the momentary reward. The rise reaches its peak around 1 s after the onset of the reward-related stimulus and starts to decline after 2 s, reaching the baseline concentration after around 4 s [66]. Taking this time course into consideration, we applied the TMS during the probabilistic reward task at the expected time of the peak dopamine concentration in the basal ganglia. However, SICI and SAI were recorded before and after our probabilistic reward task. Therefore, a decrease in SICI for the agonist muscle occurred over the time course of dopamine concentration in our study. Rosenkranz et al. [35] and Smyth et al. [36] suggested that the improvement in task performance during the early practice phase occurred through unmasking of pre-existing intracortical connections and increasing the efficacy of existing synaptic connections, including LTP mechanisms mediated by down-regulation of GABAergic inhibition. In the present study, one possible explanation for the decrease of SICI for agonist muscle could involve increasing the efficacy of existing synaptic connections for agonist muscle including LTP mechanisms. However, the role of changes in intracortical excitability is still unclear. Further research is needed to investigate the relation between intracortical excitability and reciprocal function during probabilistic reward task. Another possible explanation for the decreased antagonist excitability is that the M1 map expansion of the trained agonist muscle could potentially result in cortical competition with the surrounding muscle representations [32], [67], [68]. Several studies have suggested that M1 could be reorganized during motor skill acquisition [36], [69]–[72]. In addition, it is necessary to investigate further the changes in CoG and M1 map areas for the agonist and antagonist muscles during probabilistic reward tasks.

There was no between-probabilities difference in reaction time. One possible explanation for this is that the result of reaction time was not related to reward probability. In fact, the task of this experiment predetermined reward probability. Even if the subjects performed wrist flexion and pressed the button more quickly, the predetermined reward probability did not reflect the results of reaction time. Therefore, reward probability might not influence reaction time. Further research is needed to investigate the relation between reward probability associated with behavioral results and M1 excitability for reciprocal muscles.

In conclusion, we found that M1 excitability for agonist and antagonist muscles changed during performance of a probabilistic reward task. Our study provided evidence that relative MEP amplitudes for both reciprocal muscles before reward stimulus were the highest for 10% reward probability during the task, but relative MEP amplitudes after reward stimulus were the highest for 90% reward probability during the task. These results implied that M1 excitability for reciprocal muscles including the reward-related circuit before and after reward stimulus could be differently altered by reward probability.
